# The scotogenic contact lens: a novel device for treating binocular diplopia

**DOI:** 10.1136/bjophthalmol-2014-305985

**Published:** 2015-02-13

**Authors:** Matthieu P Robert, Fabrizio Bonci, Anand Pandit, Veronica Ferguson, Parashkev Nachev

**Affiliations:** 1 AP-HP, Service d'Ophtalmologie, Hôpital Universitaire Necker-Enfants Malades, Paris, France; 2 COGNAC-G, UMR 8257, CNRS-IRBA-Université Paris Descartes, Sorbonne Paris Cité, France; 3 Faculty of Medicine, Imperial College London, London, UK; 4 Western Eye Hospital, Imperial College Healthcare NHS Trust, London, UK; 5 Institute of Neurology, UCL, London, UK

**Keywords:** Contact lens, Field of vision, Treatment other, Visual perception

## Abstract

Binocular diplopia is a debilitating visual symptom requiring immediate intervention for symptomatic control, whether or not definitive treatment is eventually possible. Where prismatic correction is infeasible, the current standard is occlusion, either by a patch or an opaque contact lens. In eliminating one problem—diplopia—occlusive techniques invariably create another: reduced peripheral vision. Crucially, this is often unnecessary, for the reduced spatial resolution in the periphery limits its contribution to the perception of diplopia. Here, we therefore introduce a novel soft contact lens device that instead creates a monocular central scotoma inversely mirroring the physiological variation in spatial acuity across the monocular visual field, thereby suppressing the diplopia with minimal impact on the periphery. We compared the device against standard eye patching in 12 normal subjects with prism-induced binocular diplopia and 12 patients with binocular diplopia of diverse causes. Indexed by self-reported scores and binocular perimetry, the scotogenic contact lens was comparably effective in eliminating the diplopia while significantly superior in acceptability and its impact on the peripheral visual field. This simple, inexpensive, non-invasive device may thus be an effective new tool in the treatment of a familiar but still troublesome clinical problem.

## Introduction

It is widely assumed that innovation in medical treatment can only follow innovation in medical science, novel action naturally requiring novel fact. But there is a great deal of established fact—especially in physiology—whose therapeutic potential is underappreciated.

Here we apply such ‘retro-innovation’ to the management of binocular diplopia, a symptom reported by an estimated 120 000 people in the UK (data from Driver and Vehicle Licensing Agency for 2010). Where the misalignment of the eyes cannot be immediately corrected—the majority—the common approach is to remove one of the two images by occluding one eye, usually with a ‘pirate-style’ eye patch. This eliminates the symptom, but at the cost of creating another: a reduction in the visual field between 48% and 76%.^1^ Crucially, while we do not use the peripheral visual field for exploratory vision, its sharp temporal sensitivity is key to navigating in response to sudden events.^2^ Many patients find its loss unsettling, sometimes as much as the diplopia itself.^3^

**Figure1:**
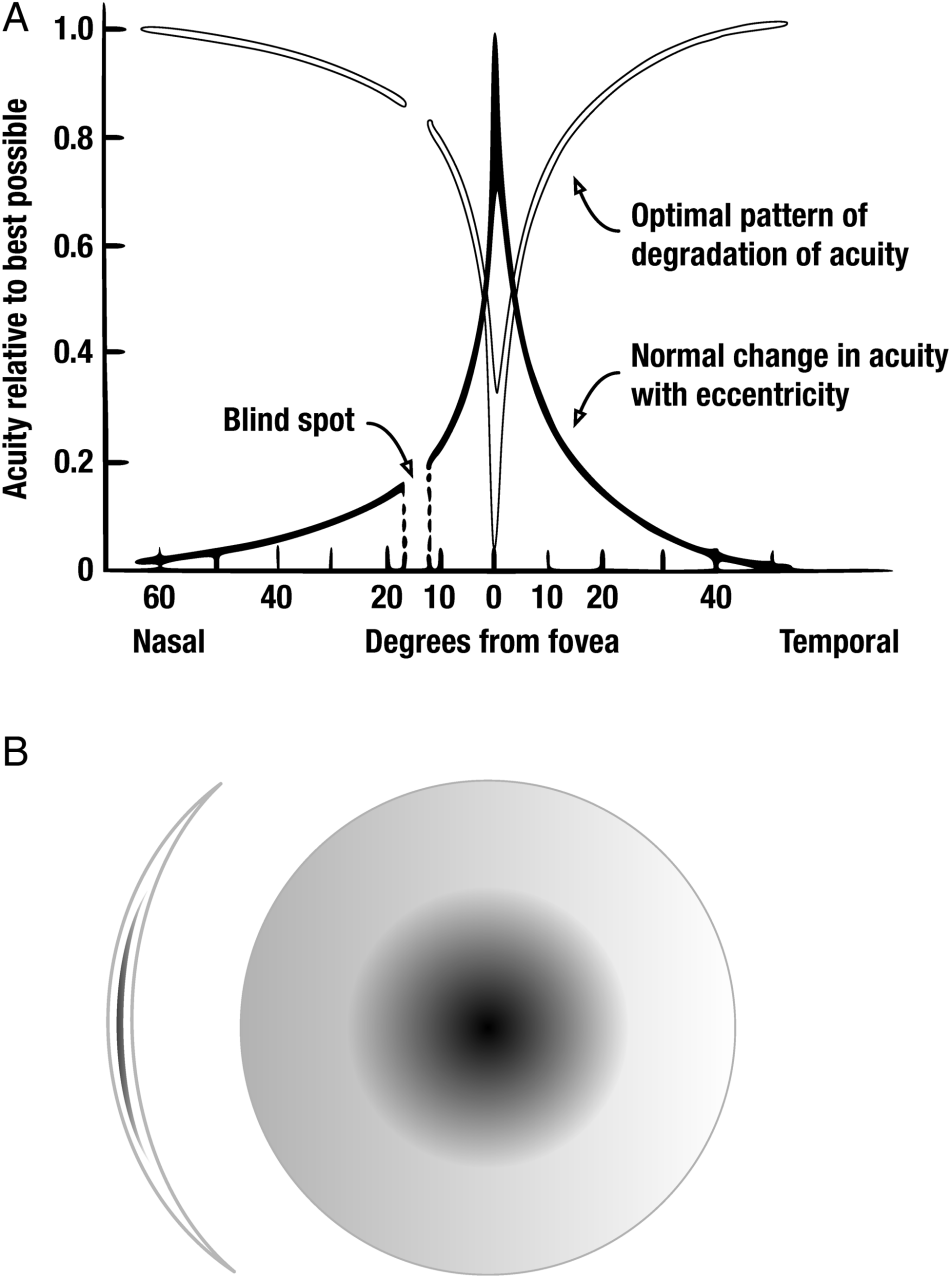
(A) Diagrammatic representation of the variation in spatial acuity across the visual field (black line) and the consequently optimal pattern of degradation of the perceived image produced by a scotogenic lens designed to abolish binocular diplopia with minimal effect on the peripheral visual field (white line). (B) Diagram of the design of a scotogenic contact lens (not to scale). Dispersant material (in our prototypes barium sulfate combined with black dye) is introduced in the substance of the lens so as to produce the optimal pattern of degradation of acuity across the visual field described in (A).

The high temporal sensitivity of the peripheral field, however, is offset by low spatial sensitivity.^4^ ^5^ Since diplopia is principally a percept in space, not time, it typically does not occur in the periphery, for the spatial detail of the perceived image is too low there. The same is generally true of the rarer symptom of confusion: the perception of two different overlapping objects owing to binocular misalignment. To eliminate diplopia by occlusion, one therefore does not have to obscure the whole field but only to *degrade* it in a pattern that is the inverse of the variation in spatial acuity across the visual field: in short, one has to create a central scotoma in one eye (figure 1A). The result, predictable from the well-known physiology of vision, is abolition of diplopia with minimal impact on the peripheral field. Current occlusive contact lenses and implantable lenses either abolish vision in the eye altogether or degrade vision in a way that does not target this theoretical optimum.^6^

Though seemingly complex, this effect is readily achieved with the aid of a contact lens that simply blurs the image in the corresponding pattern with the aid of dispersant distributed in a radially reducing concentration (figure 1B). Here, we report the design and evaluation of a prototype of such a ‘scotogenic’ lens (coined from the Greek σκότος (darkness)+γένεσις (creation)) in 12 normal participants with artificially induced diplopia and 12 patients with symptomatic diplopia of a disparate variety of causes, compared against conventional eye patching.

## Methods

We created a prototype scotogenic lens by precipitating barium sulfate within the substance of a soft hydrolens contact lens in the form of a disc with radially thinning edges of an approximate overall diameter of 7 mm. Dye was added to the precipitate so as to achieve the appearance of a black pupil. The parameters of the lens were base curve 8.60 mm and diameter 14 mm, a fitting that suited all participants in the study. The base lenses were manufactured by Cantor and Nissel (http://www.cantor-nissel.co.uk/) and the prototypes were prepared by SCL Contact Lenses (http://www.sclcontactlenses.com/).

To evaluate the performance of the lens, we recruited 12 healthy adult volunteers and 12 adult patients with binocular diplopia attending the ophthalmology service at Imperial College Healthcare National Health Service Trust. The former were unselected except for freedom from any significant ophthalmological disease. The inclusion criterion for the latter was the presence of symptomatic binocular diplopia of any cause; exclusion criteria were inability to give informed consent, contraindications to soft contact lens use and inability to complete a questionnaire. The characteristics of the patients are summarised in online supplementary table S1. In most cases, diplopia was secondary to an oculomotor palsy or restriction, so that the deviation and impairment varied considerably according to gaze position. All patients had previously been comprehensively evaluated by an ophthalmologist (VF) and found to be free from contraindications to soft contact lens use. In addition, all participants were evaluated immediately before the testing session by an optometrist (FB), including a clinical assessment of visual acuity and motility and slit lamp examination of the ocular surface. Diplopia in the healthy participant group was artificially induced with the aid of a Fresnel prism applied to one eye, generating a 10° full-field misalignment.

The evaluation process involved wearing either the scotogenic lens or a black occlusive patch on one eye—each for a period of 30 min of free reading and room exploration, with the order randomised across participants—followed by completion of a questionnaire where effectiveness in abolishing the symptom, comfort, aesthetics, and overall success were each rated on a 10-point scale (0 poor, 10 perfect, see online supplementary material). In addition, automated Estermann perimetry was performed on the normal participants in each test condition with a Humphrey visual field apparatus.

Questionnaire scores and the results from the Estermann perimetry were analysed by standard two sample Kolmogorov–Smirnoff (K–S) tests, quoting asymptotic p values. We used K–S because it makes no assumptions about the nature of the underlying distributions and is maximally conservative, minimising the risk of type II errors.

## Results

**Figure 2:**
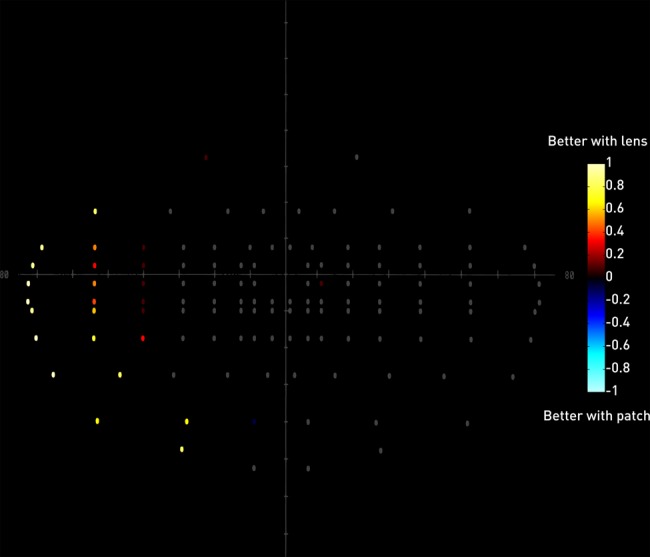
Heatmap depicting the mean difference between the two conditions for each location of the Estermann perimetry test. For each participant, a location was given a 1 if successfully detected with the lens but not the patch, and −1 if detected with the patch but not the lens. Mean values close to 1 therefore indicate superiority of the lens across the group (red to yellow range), and values close to −1 the converse (blue to cyan range). Locations without a difference are given in grey. Note that where there is a difference the lens is substantially superior for all locations but one.

In the normal participant group (table 1), both methods were equally effective in abolishing binocular diplopia and in comfort, but the lens was rated significantly superior in aesthetics and overall. Performance on Estermann perimetry was significantly superior with the lens, further illustrated in a heatmap of the mean difference between the two conditions at each field location (figure 2).

**Table 1:** Questionnaire and Estermann scores for the group of normal participants with artificially induced diplopia

Test	Occlusive patch (means)	Scotogenic lens (means)	Significance (K–S test), p value
Symptom control	8.42	8.75	0.991
Comfort	6.50	6.17	0.990
Aesthetics	2.33	8.75	**<0.001**
Overall assessment	6.42	8.67	**0.005**
Estermann score	14.92 missed locations	0.92 missed locations	**<0.001**

Bold represents significance at the p=0.05 level.

K–S, Kolmogorov–Smirnoff.

In the patient group (table 2), both methods were identically effective in abolishing binocular diplopia, but the lens was significantly superior to all other measures.

**Table 2:** Questionnaire scores for the group of patients with diplopia

Test	Occlusive patch (means)	Scotogenic lens (means)	Significance (K–S test), p value
Symptom control	9.73	9.27	0.985
Comfort	4.36	7.45	**0.047**
Aesthetics	2.00	9.45	**<0.001**
Overall assessment	4.18	8.54	**<0.001**

K–S, Kolmogorov–Smirnoff.

## Discussion

We show that it is possible to eliminate diplopia by selective degradation of the image in one eye *without* significantly impairing peripheral vision, thereby eliminating a major negative consequence of occlusion. The comparison with eye-patch occlusion—the most effective means of eliminating diplopia across the entire field—maximised the stringency of the test.

Preserving peripheral vision benefits all patients, but especially those with pre-existing field defects in whom full monocular occlusion may disqualify from important activities such as driving.

Though the extent of image degradation will interact with the size of the pupil, we did not observe functionally significant consequences of such variation—perhaps because of the graded boundary—despite considerable variability in pupil size. In any event, the direction of variation is favourable to the effect we are seeking, for a more pronounced effect is desirable where illumination is higher and therefore acuity greater. The graded boundary will also minimise the perception of contact lens movement. Clinical embodiments of the device may allow a choice of scotoma sizes, allowing tailoring for this and other effects such as interindividual variability in Panum's fusional area.

Though here we use a dispersant material—following occlusive lens technology—essentially identical perceptual effects can be achieved by diffraction, without altering the substance of the lens. Even so, the much smaller amount of dispersant material used in our prototypes compared with fully occlusive lenses greatly minimised any adverse impact on oxygen permeability.

## Supplementary Material

Web supplement

Web table
