# Supplementary material for: The scotogenic contact lens: a novel device for treating binocular diplopia
Source: Br J Ophthalmol. 2015 Feb 13;99(8):1022–4. doi: 10.1136/bjophthalmol-2014-305985 (PMC4518759; doi:10.1136/bjophthalmol-2014-305985)
Supplement: Web supplement [file bjophthalmol-2014-305985-s1.pdf]

## Participant evaluation procedure

The primary outcome measure of the study was a scored, individual evaluation by each participant based on a questionnaire (reproduced overleaf). As befits a participant-led evaluation, the process was designed to be as ecologically valid as possible within the constraints of the study setting. For each therapeutic option (patch or contact lens), in order randomised across participants, participants spent 30 minutes engaged in free reading and room exploration. Participants were free to use whatever visual acuity aids they normally used (spectacles or contact lenses), except for corrective contact lenses in the treated eye (for that was either occupied by the lens or patched).

The reading material included popular magazines and daily newspapers containing text printed in the range of typeface sizes in popular use in standard publications of this nature. The material included images and graphics with a similarly representative range of spatial properties. Participants were encouraged to examine the material as they would naturally do if they were interested in its contents, both text and images, and to characterise their ability to do this as best they could.

The room exploration component involved navigating through the examination room and adjacent clinic waiting area, including looking outside through the windows. All spaces were lit at the conventional level for public spaces of this nature, and contained a range of static and dynamic objects representative of such environments. Participants were encouraged to explore the spaces, including looking outside through the windows, as if they were required to describe them in detail later. As in the reading component, participants were encouraged to reflect on their ability to do this under the two test conditions. The room exploration component included navigation to, and exploration of, a head-sized mirror so as to allow the participant to comment on aesthetic impact.

Participants were free to allocate time to each component as they felt its demands justified, and as they were able to characterise its aspects to their satisfaction. They were asked to fill in the questionnaire for both therapeutic options at the end of the second (and last) therapeutic option. Participants read the questionnaire and the rating scales were explained to them orally, so as to confirm that they understood them. They were encouraged to be as sincere and objective in their evaluation as possible.

## Questionnaire

## Scotogenic contact lens

1. Please score out of 10 (0 useless – 10 flawless) the performance of the two methods in reducing the perception of double vision.

| | |
|---|---|
| Eye patch | |
| Contact lens | |

2. Please score out of 10 (0 disfiguring – 10 invisible) your impression of the aesthetic impact of the two methods.

| | |
|---|---|
| Eye patch | |
| Contact lens | |

3. Please score out of 10 (0 uncomfortable – 10 comfortable) your impression of the comfort of the two methods.

| | |
|---|---|
| Eye patch | |
| Contact lens | |

4. Please score out of 10 (0 unsuccessful – 10 successful) your impression of the general success of the two methods in improving the symptom.

| | |
|---|---|
| Eye patch | |
| Contact lens | |

5. Please give any comments you think may be relevant.

| |
|---|
| |
