# Supplementary material for: The scotogenic contact lens: a novel device for treating binocular diplopia
Source: Br J Ophthalmol. 2015 Feb 13;99(8):1022–4. doi: 10.1136/bjophthalmol-2014-305985 (PMC4518759; doi:10.1136/bjophthalmol-2014-305985)
Supplement: Web table [file bjophthalmol-2014-305985-s2.pdf]

Supplementary Table 1. Clinical characteristics of the patients taking part in the study.

Patient	Sex	Diagnosis	Corrected visual acuity	Ocular misalignment in primary gaze, in degrees
1	M	Dragged fovea syndrome	Right 6/12<br>Left 6/5	Dist: Right hypertropia, 3<br>Near: Right exotropia, 2
2	F	Thyroid eye disease	Right 6/5<br>Left 6/12	Dist: Right hypertropia, 2<br>Near: Right hypertropia, 2
3	F	Microvascular 6th nerve palsy	Right 6/9<br>Left 6/12	Dist: Right esotropia, 4<br>Near: Right esotropia, 4
4	F	Microvascular 3rd nerve palsy	Right 6/18<br>Left 6/18	Dist: Left exotropia, 6 & left hypotropia, 2<br>Near: Left exotropia, 4
5	F	Skew deviation secondary to MS	Right 6/12<br>Left 6/9	Variable left hypotropia and excyclotorsion
6	M	Traumatic 3rd nerve palsy	Right 6/5<br>Left 6/9	Dist: Left exotropia, 6<br>Near: Left exotropia, 6
7	F	Microvascular 6th nerve palsy	Right 6/24<br>Left 6/12	Dist: Left esotropia, 6 & left hypotropia, 4<br>Near: Left esotropia, 6 & left hypotropia, 4
8	F	Thyroid eye disease	Right 6/4<br>Left 6/4	Dist: Left hypertropia, 4<br>Near: Left hypertropia, 3
9	M	Midbrain stroke	Right 6/6<br>Left 6/6	Dist: Right exotropia, 2<br>Near: Right exotropia, 6
10	M	Unknown	Right 6/5<br>Left 6/18	Dist: Left exotropia, 6<br>Near: Orthotropia
11	M	Left orbital tumour	Right 6/5<br>Left 6/12	Orthotropia, but severe limitation of left eye motility
12	F	Traumatic 4th nerve palsy	Right 6/6<br>Left 6/6	Dist: Right hypertropia, 2<br>Near: Right hypertropia, 1
